# The rapid increases in microplastics in urban lake sediments

**DOI:** 10.1038/s41598-020-57933-8

**Published:** 2020-01-21

**Authors:** Mingtan Dong, Zejiao Luo, Qingfeng Jiang, Xinli Xing, Qiaoqiao Zhang, Yue Sun

**Affiliations:** 1 School of Environmental Studies, China University of Geosciences, Wuhan, China; 2 School of LiSiguang, China University of Geosciences, Wuhan, China; 3 School of Geographic Science, Nantong University, Nantong, China

**Keywords:** Environmental monitoring, Geochemistry

## Abstract

Microplastics have received widespread attention as an emerging global pollutant. However, the research on the abundance and characteristics of microplastics entering the environment throughout history has been limited. Meanwhile, the determination of the start of the Anthropocene is important because humans have become a vital force affecting the environment and Earth surface processes. It is unclear whether the plastic can be used as an artefact to indicate the start of the Anthropocene. In this study, combined with ^137^Cs, ^210^Pb, and spherical carbonaceous particles (SCP) high-resolution chronology, a microplastics-time curve was established by using the sedimentary record from an urban lake in Wuhan city. The microplastic abundance increased from 741 items·kg^−1^ to 7707 items·kg^−1^ over the past 60 years. The microplastics were mainly fibres and composed of polyester and rayon polymers, which indicated that the microplastics most likely originated from textiles. The surfaces of the older microplastics were rough and weathered with many absorbed elements. Microplastics are similar to fossils belonging to the Anthropocene, and may be used as an indicator. The comparison of microplastic-time curves in different records on a global scale will be necessary in the future.

## Introduction

Plastic is an important invention of the 20th century and has significantly modified our way of life, but several properties of plastic, including being non-degradable and difficult to sort and recycle, also result in great harm to the environment. In 2018, the world’s total plastic production reached 359 million tons, but only 47.1% of waste plastics in Europe were properly disposed of recycling, energy recovery and landfill^1^. After plastic enters the environment, it is broken down into pieces, and the pieces with sizes less than 5 mm are called microplastics (MPs)^2^. MPs are considered an emerging pollutant^3^ because they have strong abilities to become enriched in heavy metals^4,5^ and persistent organic pollutants (POPs)^6,7^ and can affect the growth of organisms^8^. Since 2013, extensive data have been accumulated on the spatial distribution of MPs in coastal areas^9,10^, oceans^11,12^, rivers^13,14^, lakes^15,16^, sediments^17,18^ and soil^19,20^. MPs have been found in remote areas^21,22^, which confirmed that MPs could be transported over long distances via the atmosphere^21^ and ocean currents^12,15^, which makes MPs an extensive global pollutant. However, it is unclear whether the characteristics of MPs change over time and there is no information on the abundance and types of MPs that have entered the environment throughout history. Ostle *et al*. reconstructed the changes in the contents of macroplastics in the North Atlantic seawater over the past 60 years through the recording of entanglement events caused by plastics during the Continuous Plankton Recorder survey^16^. Chiba *et al*. summarized the records of macroplastic debris found in the deep sea over the past 30 years^23^. In the absence of long-term actual observation records, understanding and evaluating MPs pollution to select a suitable sedimentary record to obtain information on MPs contamination throughout history is of great value.

Over the past century, plastics have been invented and released into the environment and a series of environmental problems have also emerged, including increased CO_2_ concentrations and global warming^24^, increased extreme weather events such drought^25^, forest reductions^25^, accelerated species extinction^26^, and the introduction of POPs such as organochlorine pesticides into the environment^27,28^. All of these impacts have signified that human beings are a significant force affecting the Earth’s ecological environment^29^ and profoundly altering the planet. Under this background, the term “Anthropocene” was proposed by Crutzen^30,31^ and has received extensive attention over the last decade. However, geologists and geographers have disagreed about the time when the Anthropocene began^32,33^. At a recent conference of the International Commission on Stratigraphy, members of the Anthropocene Working Group (AWG) decided to establish the Anthropocene as a new epoch and voted to use the mid-twentieth century as the beginning of the Anthropocene^34^. The group will identify a global boundary stratotype section and point (GSSP), which is commonly known as a “golden spike”, in the following years. Plastic is widely used as a synthetic compound by humans and is also believed to indicate the beginning of the Anthropocene^8,27,33,35^, but there is still lack of sufficient studies about (micro)plastics abundance and characteristics versus time.

We selected the second-largest urban lake in China, Donghu Lake, as the study area and collected a sediment column. The MPs with a detection limit of 100 μm versus time were studied by the ^210^Pb chronological data, Raman Spectrometer identification and scanning electron microscope. The changes in MPs abundance, length, colour, polymer type, and surface features over time were studied, and the possibility of using MPs as an indicator of the Anthropocene was discussed.

## Results

### The establishment of the time sequence

**Figure 1:**
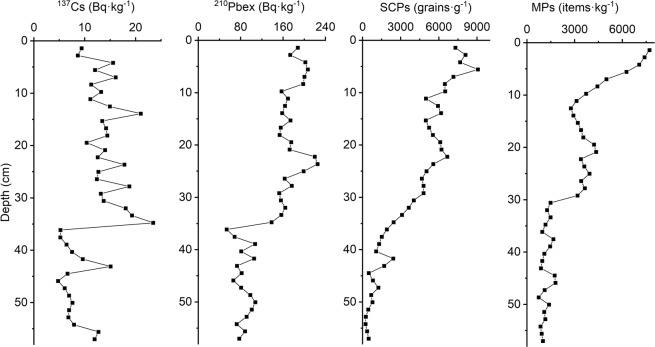
^137^Cs activity, ^210^Pbex activity, SCPs and MPs versus depth.

**Figure 2:**
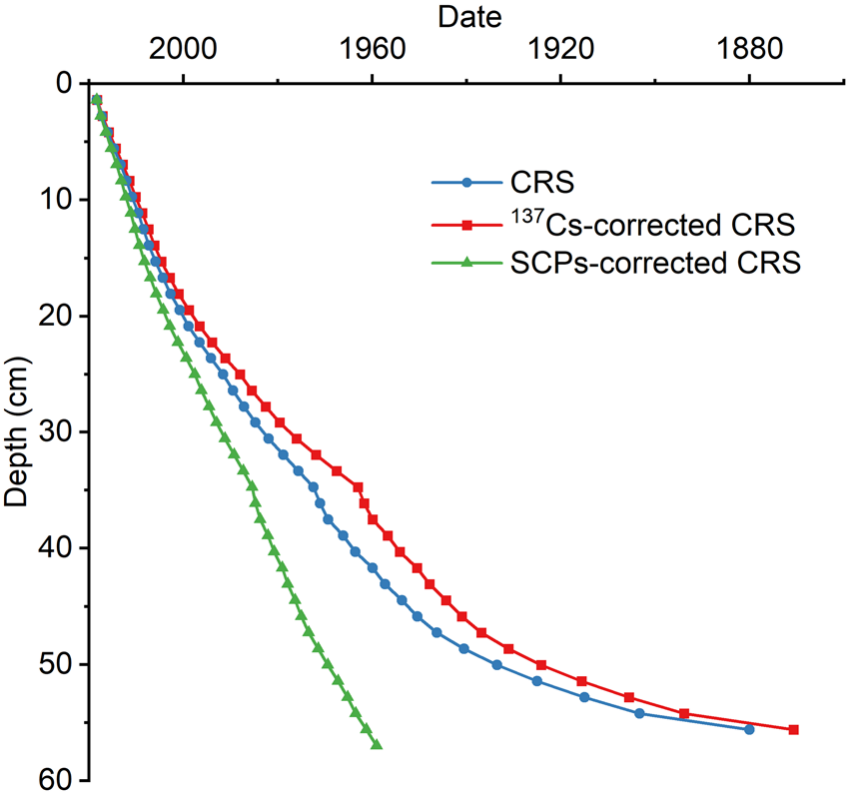
Comparison of dating results derived from alternative ^210^Pb models in the sediment cores.

The variations in ^137^Cs, ^210^Pbex, SCPs, and MPs with depth are given in Fig. 1. The CRS model was used to calculate the data, and the results are shown in Fig. 2. The activity-depth curve of ^137^Cs did not conform to the typical unimodal distribution. The maximum value of 23.4 Bq·kg^−1^ appeared at 34.8 cm in the middle of the sedimentary core, but it was uncertain whether this region represented the peak of the 1963 nuclear boom. The large fluctuations in ^137^Cs at the bottom of the sedimentary column indicated that the bottom of the sediment core underwent a large disturbance in the historical period. At the same time, ^137^Cs never fell to zero, indicating that the sediment should have originated after the nuclear tests in the 1950s. When the ^137^Cs maximum value was used as the 1963 time marker to correct the CRS, the resulting data were older (Fig. 2), which did not satisfy the condition that the ^137^Cs content should be zero before the 1950s. The ^137^Cs-corrected CRS model was not suitable for calibration of ^210^Pb in this sediment core.

SCPs were also detected at the bottom of the sediment. Based on the history of coal power generation in the study area^36^, the bottom sediment was deposited after the 1950s^36^, and the result was consistent with the ^137^Cs time marker. The bottom sediment was set to 1959, and the entire sedimentary column data were corrected based on this time marker. The SCP-corrected results that were obtained (Fig. 2) are well fitted to the ^137^Cs activity. According to the time of invention and use of plastic products, it was believed that the SCP-corrected results were reliable.

### MPs abundance versus time

**Figure 3:**
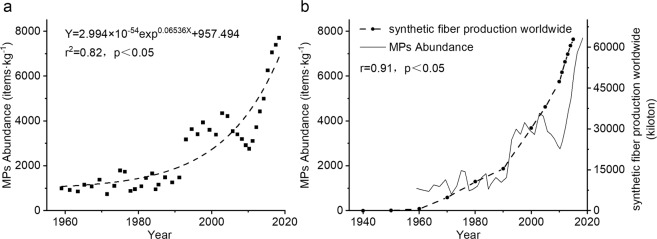
The abundance of MPs versus time and comparison with synthetic fibre production worldwide. The increase of microplastic abundance is significantly correlated with the exponential function and synthetic fibre production worldwide over the same period (1959–2018). The synthetic fibre production worldwide was linearly interpolated and the correlation coefficient between it and the microplastic abundance was calculated (**b**). World synthetic fibre production data was from Statista^61^.

The MPs abundance-time curve was established from 1959 to 2018 (Fig. 3). The MPs abundance ranged from 741~7707 items·kg^−1^; the minimum value appeared in 1971, and the maximum abundance was located in the outermost layer of sediment, which appeared in 2018. The MPs abundance curve in stages with the boundaries in 1993 and 2010. In 1993, and the MPs abundance before and after was doubled. In 2010, the MPs abundance changed from a decrease to an increase. Since1993, the MPs abundance has maintained a rapid growth trend. Two periods of rapid growth occurred from 1993~2004 and 2010~2018. From 1959 to 1993, the MPs abundance was at a low level of 741~1797 items·kg^−1^, and it exhibited conspicuously irregular fluctuations. From 1993 to 2010, the MPs abundance increased significantly from 741~1797 items·kg^−1^ to 2764~4349 items·kg^−1^. The MPs abundance changed in a zigzag manner, but the overall trend was still rising. From 2010 to 2019, the abundance of MPs increased from 3112 items·kg^−1^ to 7707 items·kg^−1^. MPs abundance-time changes were exponentially and were significantly correlated with synthetic fibre production worldwide in the same period. The MPs abundance-time curve in this study was consistent with the multidecadal increase in plastic particles in coastal ocean sediments reported by Brandon *et al*.^37^.

### MPs length, colour, and polymer type versus time

**Figure 4:**
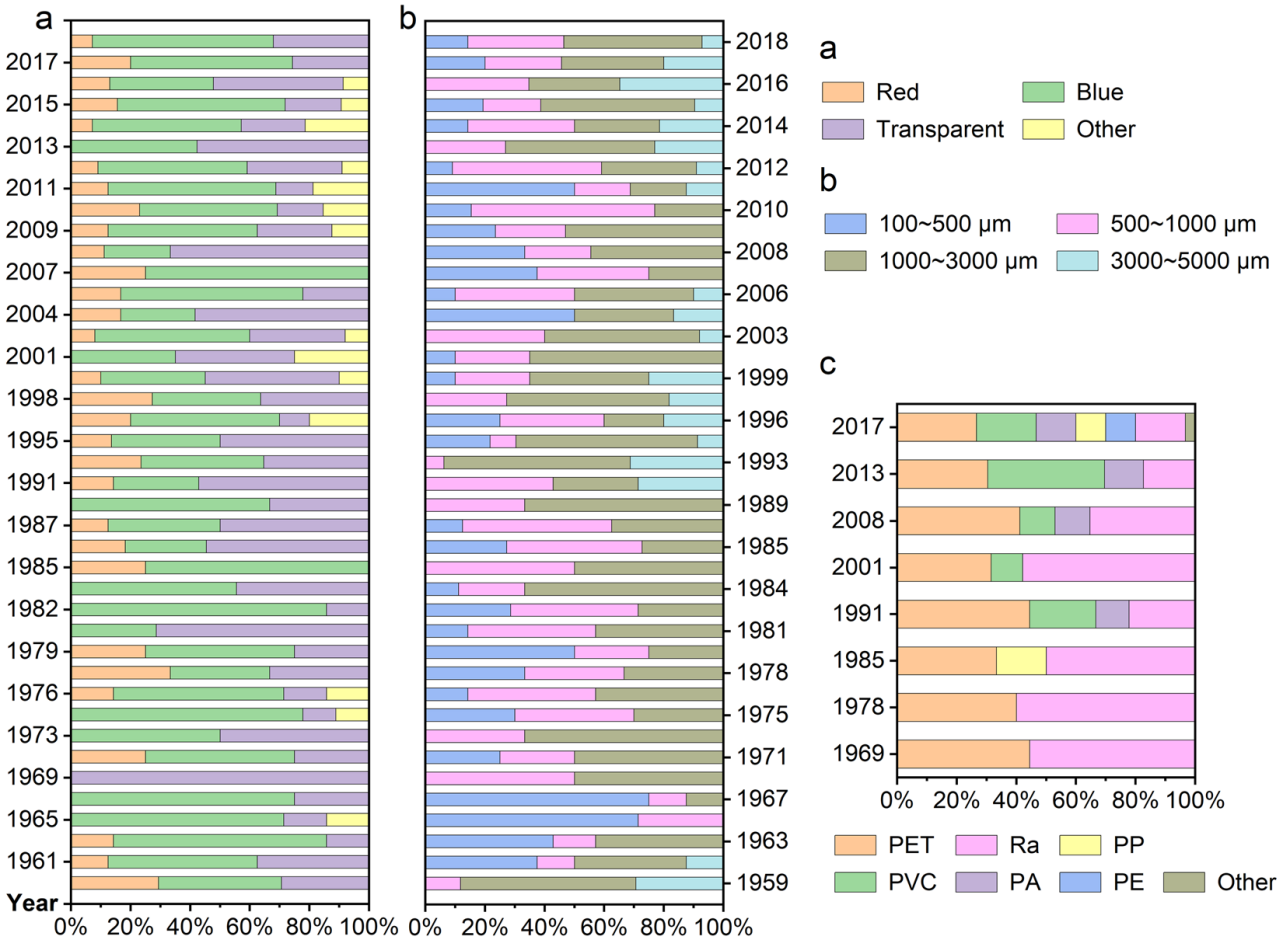
The colours (**a**), length(**b**) and polymer types (**c**) of MPs versus time.

The MPs were all composed of fibrous MPs and long strips, with no debris or pellets. Li *et al*.^38^ also found that fibrous MPs were the major components of the MPs in the lake sediments of the middle and lower reaches of the Yangtze River Basin. Therefore, the length instead of the particle size was used to classify the MPs. In general, the colours of the MPs were mainly transparent and blue (Fig. 4a), and the lengths of the MPs (Fig. 4b) were mainly 500~1000 and 1000~3000 μm. The percentage of large MPs (3000~5000 μm) and other colours in the upper sediment were higher than in the lower.

The polymer types changed significantly with time (Fig. 4c). The MPs detected in the upper layer sediments had the largest variety of polymer types, while the MPs detected in the lower layer sediments were all made up of polyester (PET) and rayon (Ra). The MPs in the sediments were mainly PET and Ra (Fig. 4c) fibres. This result confirms that the MPs in the historical period also most likely originated from textiles, because studies have shown that 1900 microfibres could enter the environment when washing an adult garment^39^, and this number was increased to 640,000 to 1,500,000 items·kg^−1^ in the latest research^40^.

### MPs surface features versus time

**Figure 5:**
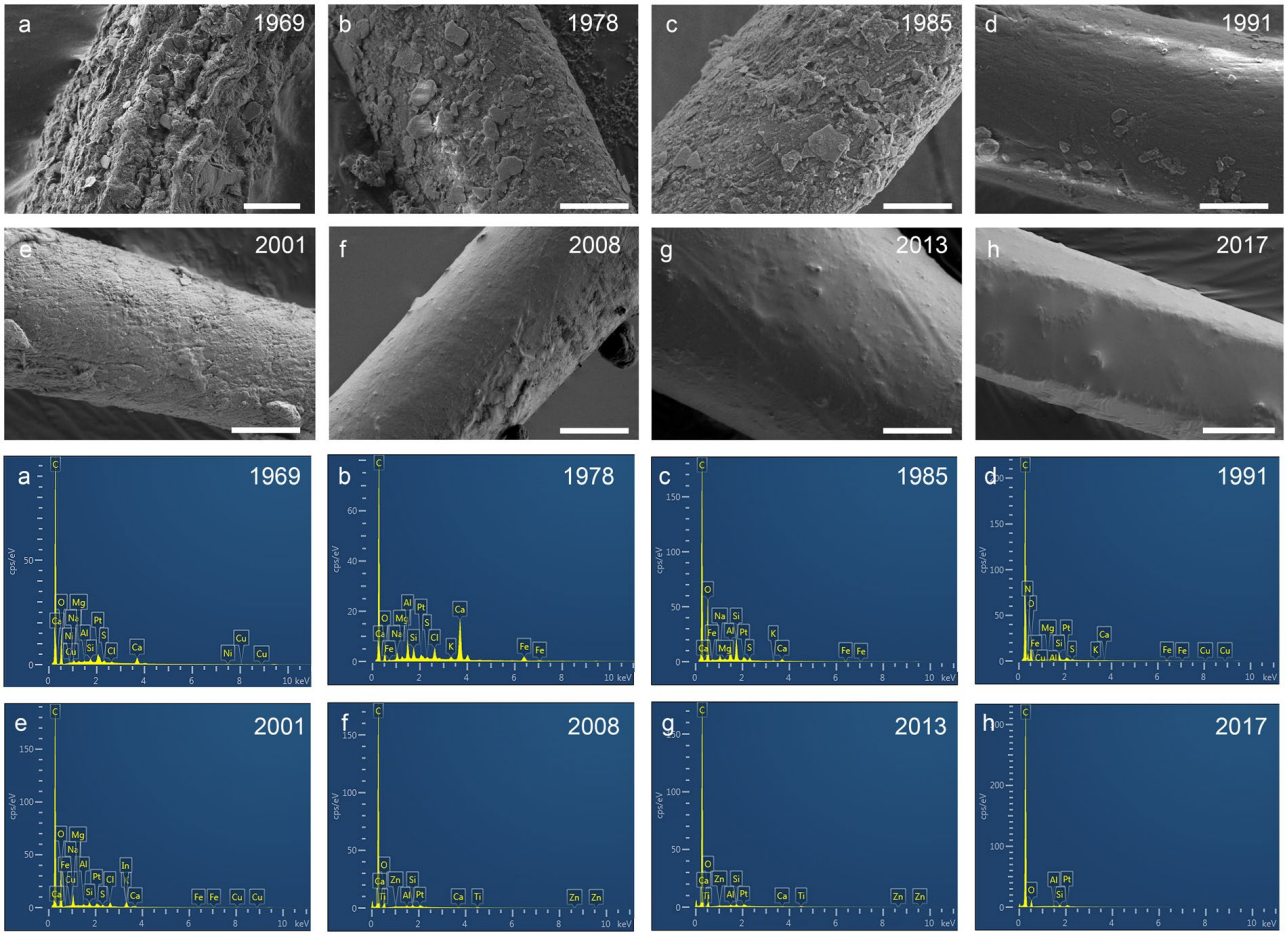
The surface feature and element composition of MPs versus time (lowercase letters correspond one-to-one). Scale bars were 5 μm in figure b~h but 10 μm in figure (**a**). The surface of fibrous MPs extracted from older sediment was rough and irregular (**a**,**b**,**c**), and the surface of MPs extracted from younger sediment was smooth (**g**,**h**).

The study of the surface feature of MPs changes with time, showing that in the older sediment core, the surfaces of fibrous MPs were rough and irregular (Fig. 5a–c), and the surface roughness of the MPs gradually decreased from the bottom to the top of the core. In the youngest sediments, the MP surfaces were very smooth (Fig. 5g, h) with no visible trace of weathering. The fibrous MPs with more weathering in the older sediments had more types of elements on the surface (Fig. 5a–d), while the MPs in the younger sediments had merely five types of elements on the surface (Fig. 5h). The weathering of MPs in the environment may affect the adsorption of elements.

## Discussion

### The uncertainty of time sequence

In order to easily find and separate MPs, the urban lake rather than lakes in the remote area was chosen as the study area. However, this had caused great potential disturbances to lake sediments because of the frequent human activities such as dredging, prospecting, tunnel construction, etc., consequently, brought the uncertainty of date results.

The CRS model assumes the sediment of lakes is mainly originate from the atmospheric deposition^36^. Although there is no river remittance in Donghu Lake, this assumption may be not corresponding with the sediment character of Donghu Lake with the consideration of frequently human activities, urban construction and expansion surrounding Donghu Lake. In this sediment core, the CRS data result was not consistent with the appearance of ^137^Cs, MPs and SCPs detected at the bottom of the core. If the maximum value of ^137^Cs at 34.8 cm was used as the 1963 time marker to correct CRS (Fig. 1), the corrected result would be consistent with the CRS (Fig. 2), but it also cannot explain the appearance of ^137^Cs, SCPs and MPs (Fig. 1) at 57 cm.

^137^Cs, SCPs and MPs have chronological meaning, and the data results need to be consistent with them. The historical record of coal power generation in this area indicated that the SCPs appeared after 1958. If any middle sediment layer was used as the time marker, the result would be older and not in accordance with the test results of ^137^Cs and MPs (Fig. 2). The maximum value of ^137^Cs at 35.7 cm and sub-maximum value at 43.1 cm shown that the bottom sediment might be younger than in 1963. Thus, we believed 1959 should be the oldest time of the bottom sediment combine with the above discussion, so we set the bottom sediment to 1959 and corrected the data for the entire sedimentary column.

According to the SCPs-corrected CRS data (Fig. 2), the average deposition rate at the sampling site reached 0.95 cm·year^−1^, which is generally consistent with the deposition rates in other lakes in the middle and lower reaches of the Yangtze River^36^. Yang *et al*.^41^ (2004) measured a deposition rate of 0.87 cm·year^−1^ in the same lake, but the sampling site of this study was closer to land than the sampling site investigated in Yang *et al*. Therefore, the deposition rate of 0.95 cm·year^−1^ was reasonable.

### MPs: a potential indicator of anthropocene

In stratigraphy, the first appearance of a fossil is usually used as a sign of the beginning of a geological era, and the GSSP is usually established using first appearance datum (FAD)^42^. The invention and extensive use of each plastic polymer are similar to the birth and prosperity of each new species, and plastic may serve as a new fossil and should have an indication sense on the stratum for the future geologist.

The crucial signs of entering the Anthropocene in geological history around the 1950s were the use of synthetic chemical pesticides and the discharge of pollutants^27^. The use of different chronological techniques combined with analytical chemistry techniques to analyse chemical signals in different natural sedimentary records enabled us to reconstruct historical emission trends. The chemical records that have been studied mainly include records of Dichlorodiphenyltrichloroethane (DDT), Polycyclic aromatic hydrocarbons (PAHs), Polybrominated diphenyl ethers (PBDEs), Polychlorinated biphenyls (PCBs), and heavy metals such as Pb, Hg, and Cr. MPs are different from these chemical signals as they can be observed directly under a microscope after extraction from the sediment records and appropriate pretreatment. This procedure may be more in line with the habits of traditional geologists. Different forms of PAHs may originate from forest fires^43^, and elements such as Pb and Hg have geochemical background values^44^; however, MPs can be produced by only human activities. MPs are more in accordance with the definition of the Anthropocene as an artificial object and can be used to explain the impact of humans on the environment.

SCPs can also be used as a globally synchronous stratigraphic marker to indicate the Anthropocene^45^. Compared with SCPs, MPs have different polymer types. Each polymer type was invented at different times. It is possible to achieve an accurate division of the stratum by more detailed stratification and identification of polymer types. The first appearances of different MPs polymer types detected in this research are shown in Fig. 4, and these times appeared after the invention of these polymers^46^. However, because of the lack of records on the production and use of plastics in this study area, we were unable to compare the first appearance time in this sediment core with the actual record.

### Prospects: what we need to consider

MPs could be a potential indicator of the Anthropocene because it is numerous, widely distributed and proven to be transportable over long distances^21^, but there are still some problems that need to be considered. First, in this study, we are unable to obtain a long enough sediment column. Therefore, it is not possible to differentiate the Holocene-Anthropocene boundary because of the absence of MPs information early before the 1960s. Brandon *et al*.^37^ established a reliable sediment chronological sequence and studied the plastic from 1834 to 2009. However, the detection of plastic in sediments from 1834 to 1945, which was explained to sample contamination and removed as a ‘baseline’ of the pre-industrial period, made the results seem to be uncertain. Thus, the establishment and comparison of (micro)plastic-time curve on a large-scale is necessary and important for evaluating the possibility of whether (micro)plastic could be one indicator of the Anthropocene. Secondly, studies show that the proportion of small particle size (a few microns to submicron) MPs in the environment is significantly higher than the MPs of hundreds micron^47,48^ and the smaller MPs are more easily vertically migrate^49^ in soils. Therefore, it is essential to focus on small-sized MPs in sediment and carefully evaluate the potential vertical migration of MPs in further studies. Thirdly, the persistence of (micro)plastic in the environment needs to be further evaluated because MPs were apparent weathered in about 60 years (Fig. 5). It is unclear whether the weathering of MPs is a process merely physical-chemical or with microbial participation. Roager and Sonnenschein^50^ reported the degradation of marine plastic debris with the colonization of bacteria. The persistence of (micro)plastic will affect the effectiveness of using MPs as a potential indicator of the Anthropocene in the future.

## Methods

### Study area and sample collection

**Figure 6:**
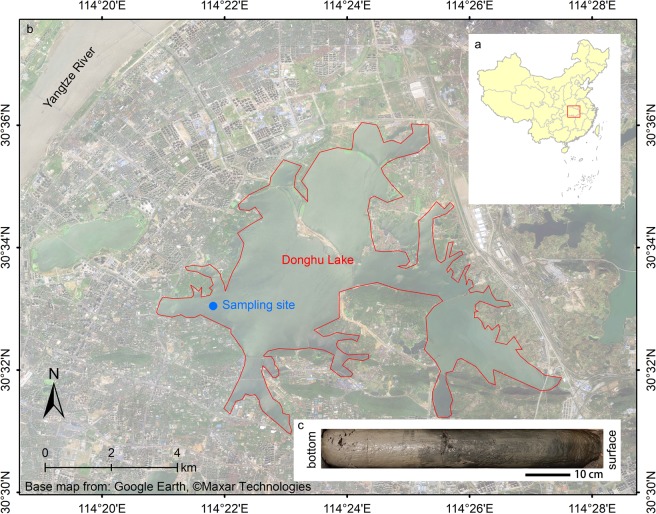
Location of the study area (**a**) and sampling site (**b**) and sediment core (**c**). Donghu Lake is surrounded by Wuhan city. It was once the largest urban lake in China, but with the expansion of Wuhan city, it was displaced by another lake in Wuhan city in 2014. The core was made by the muddy sediment without lamination or annual varves. The remote sensing base map was from Google Earth, Maxar Technologies, and the figure was created by ArcMap 10.6.

The sampling site (114°21′51.27″E, 30°32′57.58″N) is located in Donghu Lake (Fig. 6a, b), Wuhan City, Hubei Province, China. Donghu Lake is the second-largest urban lake in China, with an area of approximately 33 km^2^, average water depth of approximately 2.8 m and a maximum water depth of approximately 3.1 m^51^. Over the past 60 years, because of the expansion of Wuhan city and frequent human activities, Donghu Lake has been the collection area for household, industrial and agricultural wastewater, which allow for the sediments of Donghu Lake to be used as a possible record of the MPs produced by human activities throughout this historical period.

In May 2019, sediment cores were collected by a piston gravity sampler. A total of 41 samples were obtained by cutting a 57 cm column at a 1.4 cm interval in the field. This 57 cm column was the longest core we could collect (Fig. 6c). The water-sediment interface was ensured to be clear when sampling to ensure no disturbance to surface sediments. The sampling point was approximately 300 m away from the shore to avoid disturbance to the sediment by human activities as far as possible.

### MPs extraction, visual identification and quantification

The oil extraction protocol proposed by Crichton *et al*.^52^ was used for the extraction of MPs, and the recovery rates were re-calibrated to verify the reliability of the results. First, 15~20 g (wet weight) of sediments were weighed in a 250 mL Erlenmeyer flask. Second, approximately 5 mL of oil (Corn oil, Longevity Flower, China) and 100 mL of water were added to the Erlenmeyer flask, which was shaken for approximately 30 seconds and allowed to settle for approximately 20~40 minutes. Then, the supernatant was emptied onto a millipore filter (Nylon, 5 μm pore, 47 mm diameter, JINTENG, China) and filtered using a vacuum filtration set up. The above process was repeated three times. Finally, the filter was rinsed with absolute ethanol (Analytical purity, Sinopharm Chemical Reagent Co., Ltd, China) to remove the interference of residual oil from the MPs for Raman identification. The millipore filter was removed and transferred to a storage box.

Recovery rate experiments were performed using sediments collected from the same location prior to processing the actual samples. Four polymer types, commercial and food-grade plastics of polyvinyl chloride (PVC), polyethylene terephthalate (PET), polypropylene (PP) and polystyrene (PS), were selected as standards with different colours and were triturated and sieved to 35~60 mesh (approximately 0.25~0.50 mm). A sediment sample of 15 g (wet weight) was weighed, and 10 standard MPs of each polymer type were added for a total of 40 MPs. The standard MPs were extracted according to the protocol and counted under the microscope to calculate the recovery rate. The recovery rate experiment was carried out in three groups. The total recovery was 90.8 ± 1.44%, and the recovery rates for PVC, PET, PP, and PS were 100 ± 0.00%, 90.0 ± 10.0%, 86.7 ± 11.5%, and 86.7 ± 5.77%, respectively.

The MPs were observed and counted using a stereomicroscope equipped with an electronic eyepiece (XTL165-MT, Phenix, China). The shape, colours, and length of the MPs were recorded. The limit of MPs detected was 100 μm to 5000 μm because studies have shown that visual identification was usually inaccurate in the identification of MPs with small-size^53^. Samples were stored at room temperature, but all extraction processing was completed within 14 days after sampling. Based on the stereomicroscope visual identification counting results, MPs abundance is expressed as items per kilogram dry mass of sediment (items·kg^−1^).

### Raman analysis

A confocal laser Raman spectrometer (LabRAM HR Evolution, HORIBA, Japan) equipped with a 532 nm and 633 nm laser was used to identify the MP polymer type. From 41 sediment samples, 8 samples were selected, and all the MPs in these 8 samples were identified one at a time. Spectra were collected in the spectrum range of 300~3300 cm^−1^ using an acquisition time of 10 seconds with two accumulations and a 0.1~5% power filter (usually 1%) to avoid burning through the MPs. For each MP, we chose different locations and switched the 532 nm and 633 nm laser using a fast switcher to avoid fluorescence interference to obtain a spectrum of sufficient quality.

Because the Raman spectrometer was not equipped with a spectra database, all common polymer types of plastic were scanned in advance to collect their spectra under the same conditions to establish the standard spectra of plastics. A total of 19 spectra of 8 polymer types were obtained and composed of the database. The spectra of MPs in the sediment were compared to standard spectra to identify the polymer type. All Raman analyses were completed at the State Key Laboratory of Geological Processes and Mineral Resources, China University of Geosciences (Wuhan).

### SEM-EDS analysis

A scanning electron microscope (GeminiSEM 300, Zeiss) was used to obtain the surface features of the MPs. The magnification was 500~5000×, and the acceleration voltage was 3 kV. An energy dispersive spectrometer (Oxford X-MAX) was used to obtain the element mapping of the MP surface. SEM-EDS analysis was performed by Wuhan Sousepad Testing Technology Co., Ltd.

### ^137^Cs and ^210^Pb test

Approximately 30 g (wet weight) of the sediments were weighed accurately, dried at 105 °C to constant weight and weighed to calculate the water content. The dried sediments were triturated, placed in 5 mL Eppendorf tubes, and accurately weighed. The processed sediments were left for three weeks to be radioactively equilibrated for chronological testing. The samples were tested for radioactive elements of ^137^Cs, ^210^Pb and ^226^Ra using a gamma spectrometer (Ortec HPGe GWL). The counting time was 43200 seconds (12 hours), and the activities of the above radioactive elements were read at the detection spectra of 661 keV, 46.5 keV and 295 keV. The ^137^Cs and ^210^Pb test was completed at the School of Geographic Science of Nantong University.

### SCP counting

Spherical carbonaceous particles (SCPs) originate from the combustion of fossil fuels in the power industry. SCPs are very stable in sediments and do not easily migrate. SCPs can be used to correct the age of the constant rate of supply (CRS)^36^. The experimental procedure followed the improved method provided by Rose^54,55^. The SCPs in the sediments were extracted using HNO_3_, HF, and HCl. Particles larger than 8 μm were identified and counted using an Olympus BX40 microscope at 400× magnification. SCP concentrations are expressed as grains per gram dry mass of sediment (grain/g).

### Blank control

During the sampling and experiment, all materials were glassware except for the piston gravity sampler produced by Polymethylmethacrylate (PMMA). Experimenters wore cotton overalls instead of chemical fibre clothes. The Erlenmeyer flask and other vessels were covered with tin foil at all times while waiting for the filtration to complete to avoid possible entry of MPs into the container. The oil and water used in the experiments were previously filtered through 1 μm millipore filters to remove potential MPs. The visual counting of MPs was performed by two experienced laboratory technicians to maximize the accuracy of visual identification. MPs extraction was completed in the fume hood. Three sets of atmospheric blank control groups were set up in the experiment without observing MPs larger than the detection limit. In all processes and tests, parallel samples were tested for every 10 tests. The relative deviation of the parallel double samples was required to be less than 20%.

### Data calculation

The constant rate of supply (CRS) model was used to calculate the age of the sediment^56,57^. This model assumes that ^210^Pb was mainly from atmospheric deposition, and the ^210^Pb brought by the source area had little effect on its inventory. The deposition flux was constant, but the deposition rate varied with time. The time-depth relationship was obtained by integrating the specific activity of ^210^Pbex in the sediment core. However, ^210^Pb dating may be inaccurate in the relatively complex sedimentary environment in the middle reaches of the Yangtze River due to the high precipitation and high erosion intensity. Thus, the 1963 ^137^Cs time marker^58^ and the SCP time marker^59,60^ were used to correct the CRS model. The calculation and correct method were provided by Chen *et al*. (2019)^36^.

## Supplementary information

Dataset 1.
